# The Discovery of a Reciprocal Relationship between Tyrosine-Kinase Signaling and Cullin Neddylation

**DOI:** 10.1371/journal.pone.0075200

**Published:** 2013-10-04

**Authors:** Samantha F. Friend, Lisa K. Peterson, Eric Treacy, Adrianne L. Stefanski, Tomasz Sosinowski, Nathan D. Pennock, Allison J. Berger, Virginia D. Winn, Leonard L. Dragone

**Affiliations:** 1 Department of Pediatrics, University of Colorado School of Medicine, Denver, Colorado, United States of America; 2 Integrated Department of Immunology, University of Colorado School of Medicine and National Jewish Health, Denver, Colorado, United States of America; 3 Department of Obstetrics and Gynecology, University of Colorado School of Medicine, Aurora, Colorado, United States of America; 4 Barbara Davis Center for Childhood Diabetes, University of Colorado School of Medicine, Aurora, Colorado, United States of America; 5 Millennium Pharmaceuticals, Inc., Cambridge, Massachusetts, United States of America; 6 Division of Rheumatology, Colorado Children’s Hospital, Aurora, Colorado, United States of America; University of Iowa, United States of America

## Abstract

While neddylation is known to activate cullin (CUL)-RING ubiquitin ligases (CRLs), its role in regulating T cell signaling is poorly understood. Using the investigational NEDD8 activating enzyme (NAE) inhibitor, MLN4924, we found that neddylation negatively regulates T cell receptor (TCR) signaling, as its inhibition increases IL-2 production, T cell proliferation and Treg development *in vitro*. We also discovered that loss of CUL neddylation occurs upon TCR signaling, and CRLs negatively regulate IL-2 production. Additionally, we found that tyrosine kinase signaling leads to CUL deneddylation in multiple cell types. These studies indicate that CUL neddylation is a global regulatory mechanism for tyrosine kinase signaling.

## Introduction

The strength of signal generated through the T cell receptor (TCR) signaling complex controls T cell development, function, and disease thresholds [1], [2]. Elaborate signaling networks downstream of the TCR have been identified, but the mechanisms that modulate signal strength are less well defined [1], [3]. Ubiquitination is a posttranslational modification that is emerging as an important regulator of TCR signaling networks and T cell effector functions [4]–[8]. However, the contribution of ubiquitin-like modifiers, such as neural precursor cell expressed developmentally down-regulated 8 (NEDD8), to regulating TCR complex-mediated signals has not been defined. NEDD8 is best known to activate a large family of Cullin (CUL)-RING E3 ubiquitin ligases (CRLs) [9], [10]. Neddylation of the CUL structural subunit causes a conformational change that induces enzymatic activity leading to the ubiquination of target proteins [11]. Recently, CUL single-nucleotide polymorphisms have been associated with enhanced T cell function in patients with rheumatoid arthritis and increased T cell loss in HIV patients [12], [13]. These studies implicate CRLs in regulating T cell function. Thus we hypothesized that neddylation of CULs activates CRLs to modulate TCR signaling thresholds and thereby regulate T cell activation and effector function.

To investigate if neddylation regulates TCR signaling, we used MLN4924, a first-in-class, investigational inhibitor of NEDD8-activating enzyme (NAE1) [14], to block CUL neddylation in both T cell lines and primary purified T cells *in vitro*. MLN4924 is currently in phase I clinical trials for various malignancies, and nonclinical data suggests that MLN4924 induces apoptosis in tumor cells, in part by inducing DNA rereplication during S phase [14]. However, the effects of MLN4924 on TCR complex-mediated signaling and T cell function are not as fully understood. Our studies demonstrate that MLN4924 increases TCR-stimulated cytokine production with low doses of TCR stimulation and that TCR complex signaling leads to loss of CUL neddylation, thereby inhibiting CRL activity. Therefore, neddylation status alters the threshold of cellular response to TCR stimulation. Furthermore, we have shown that CUL deneddylation occurs in multiple cell types after initiating tyrosine kinase signaling, uncovering a relationship between tyrosine kinase signaling and CUL neddylation that limits CRL activity providing a mechanism for negative regulation of signaling.

## Materials and Methods

### Experimental Animals

BALB/c, SKG [8] and C57BL/6 mice were bred in-house and maintained in specific pathogen free conditions according to the guidelines of the National Jewish Health Institutional Animal Care and Use Committee (IACUC) (protocol number AS2738-12-14, approval date 1/17/12). The National Jewish Health IACUC committee approved all studies preformed on primary mouse T cells.

### Plasmids

The construct encoding TCRζ, as previously published [15], was a gift from N. S. Van Oers.

### Cell Lines

The murine T cell hybridoma MA5.8, which is deficient in TCRζ, was generously provided by J. D. Ashwell [16]. MA5.8 cells stably expressing the TCRζ chain were generated (MA5.8ζ) and cultured as described [16] with 1 mg/mL G418 sulfate (EMD Millipore).

The nonobese diabetic mouse (NOD)-derived T cell hybridomas 12-4.4, I.29 and AS150 [17], [18], the M12.C3-B:9–22(RE) B cell lymphoma line and Phoenix cells were all grown as previously published [19], [20].

C57BL/6J-TgN3Ems heterozygous mouse embryonic fibroblasts (MEFs) were kindly provided by Dr. J. Matsuda from the Mouse Genetics Core Facility at National Jewish Health, [21].

The BT549 cell line, kindly provided by Dr. H. Ford, and the HT29 cell line, kindly provided by Dr. P. Jedlicka, were all grown as published [22].

### Cell Purifications

Untouched, BALB/c and SKG total CD4^+^ T cells and C57BL/6 total CD8^+^ T cells were isolated by magnetic bead separation (Miltenyi Biotec, Auburn, CA) on LS columns (Miltenyi Biotec). Resting T cells (CD4^+^CD25^−^) were isolated from spleen and LNs of BALB/c or SKG mice using magnetic bead separation (Miltenyi Biotec), followed by depletion of CD25^+^ T cells by incubating with a biotin-conjugated antibody cross-reactive against (α-) CD25, followed by a second separation using anti-biotin magnetic beads (Miltenyi Biotec). Untouched, BALB/c, resting B cells were isolated using magnetic bead separation (Miltenyi Biotec) on LS columns.

Human umbilical cord blood was provided by the University of Colorado Cord Blood Bank. Granulocytes were first depleted from the PBMCs using the RosetteSep human granulocyte depletion cocktail (StemCell Technologies, Vancouver, BC). Primary untouched CD4^+^ T cells were then purified by magnetic nanoparticle negative selection using the EasySep human CD4 selection kit (StemCell Technologies).

### Staining for Flow Cytometry

For surface marker expression, cells were stained as per our previously published protocols [8]. Flow cytometric data was collected on a Cyan flow cytometer and analyzed using FlowJov.8.7.1 software.

### Cell Trace Violet Labeling

For proliferation studies, naïve primary CD4^+^ T cells were isolated from BALB/c mice (as described above) and labeled with 10 µM CellTrace Violet (Life Technologies), as per manufacturer instructions.

### Cell Stimulations

To stimulate both MA5.8ζ T cells and primary mouse T cells for either IL-2 ELISAs or for IL-2 quantitative real-time (RT)-PCR, cells were plated in duplicate on α-CD3 (145-2C11) coated plates with the indicated concentration of MLN4924 or OPT for 12–48 hours before harvesting supernatants for ELISA or T cells for (RT)-PCR.

To stimulate NOD-derived T cell hybridomas for IL-2 ELISAs, cells were plated either with the indicated concentrations of plate-bound α-CD3 and MLN4924, or were mixed with equal numbers of live or fixed M12.C3-B:9–22(RE) APCs with the indicated concentrations of MLN4924 for 24 hours as per published method [20].

To stimulate both MA5.8ζ T cells and primary mouse CD4^+^ and CD8^+^ T cells for immunoblot analysis, the cells were coated with 5 µg/mL of α-CD3 for 20 minutes on ice before washing twice with cold PBS. They were then incubated with goat α-Armenian hamster secondary antibody (20 µg/mL) for the indicated amount of time at 37°C before lysing.

To stimulate human cord blood CD4^+^ T cells, the cells were coated with 10 µg/mL of biotin-conjugated α-CD3 (UCHT1) and 2 µg/mL biotin-conjugated α-CD28 (CD28.2) in PBS for 20 minutes on ice, before washing twice with cold PBS. Cells were then incubated with avidin (36 µg/mL) in PBS for the indicated amount of time at 37°C before lysing.

Primary mouse CD43^−^ B cells were stimulated with goat α-mouse IgM F(ab’)_2_ (20 µg/mL) in PBS for the indicated amount of time at 37°C before lysing.

To stimulate primary mouse CD4^+^ T cells, mouse B cells, MEFs, BT549 cells, and HT29 cells with pervanadate, cells were incubated with 100 µM pervanadate [100 µM sodium orthovanadate (Sigma-Aldrich), 3.4% hydrogen peroxide (Sigma-Aldrich)] for the indicated amount of time at 37°C before lysing.

### Pharmacological Reagents

MLN4924, was kindly provided by Millennium Pharmaceuticals, Inc. 1,10-Phenanthroline (OPT) was purchased from Sigma-Aldrich.

### Antibodies and Reagents

Mouse monoclonal antibodies against CD3 (17A2), CD4 (17A2), CD25 (PC61.5), Foxp3 (FJK-16s), IL-2 (JES6-1A12), IL-2 (JES6-5H4), IFNγ (XMG1.2), IFNγ (R4-6A2), CD3 (145-2C11), CD25 (7D4), CD28 (37.51), IL-2 (JES6-1A12), were purchased from eBioscience. Human monoclonal antibodies against CD3 (UCHT1) and CD28 (CD28.2) were purchased from eBioscience. Goat antibodies that cross-react with Armenian hamster IgG and mouse IgM F(ab’)_2_ were purchased from Jackson ImmunoResearch. Rabbit antibodies against CUL1 (EPR3102Y), CUL4a/b (EPR3200), CUL2 (ab133180) were purchased from Abcam. Rabbit polyclonal antibodies against CUL3 (A301-109A) and CUL5 (A302-173A) were purchased from Bethyl Laboratories, Inc. The rabbit monoclonal antibody against NEDD8 (2745) was purchased from Cell Signaling. The mouse monoclonal antibody against actin (C4) was purchased from EMD Millipore. Goat antibodies that cross-react with mouse IgG (H+L) conjugated with horse radish peroxidase (HRP) and rabbit IgG(H+L) conjugated with HRP were purchased from Southern Biotech.

### RT-PCR

RNA was purified using the RNeasy Micro kit (Qiagen) and reverse transcribed using the iScript cDNA synthesis kit (Bio-Rad). Taqman primer and probe sets for Ribosomal Protein 18s (Rps18, Mm02601777_g1) and IL-2 (IL2, Mm00434256_m1) from Life Technologies were used with SsoFast Probes Supermix (Bio-Rad).

### iTreg Polarization

For iTreg generation, purified CD4^+^CD25^−^ cells were plated in 96-well round-bottom plates coated with the indicated concentrations of α-CD3. Cells were cultured in X-Vivo15 serum-free media in the presence of 2 µg/ml α-CD28, 5 ng/ml TGF-β1 (R & D Systems), 10 ng/ml retinoic acid (Sigma-Aldrich) and the indicated concentrations of MLN4924 at 37°C for 96 hours [23].

### shRNA Infection

For shRNA infection, the following TRC1 lentiviral supernatants, which contain viral particles with shRNA expressed off of the pLKO.1-puro expression vector, were obtained from the Functional Genomics Facility (University of Colorado, Boulder, CO): CUL1 - TRCN0000012768, CUL2 - TRCN0000012776, CUL3 - TRCN0000012782, CUL4a/b - TRCN0000012790, CUL5 - TRCN0000012796. pLKO.1-puro shRNA TurboGRP control viral supernatants were obtained from Sigma-Aldrich as a control for lentiviral infection. MA5.8ζ T cells were incubated with viral supernatant and 8 µg/mL polybrene (EMD Millipore) for 2 hours at 37°C. Two days after infection, 5 µg/mL puromycin (Sigma-Aldrich) was added for selection.

### Cytokine ELISAs

To measure IL-2, 96-well flat-bottom Maxisorp Nunc plates (Thermo Fisher) were coated with 1 µg/mL capture antibody (clone JES6-1A12; eBioscience) and the IL-2 ELISAs were then performed according to manufacturer’s instructions using 0.4 µg/mL biotinylated antibody (clone JES-5A4) for detecting IL-2. Sample IL-2 concentration was determined using recombinant IL-2 (eBioscience) as a standard.

IFNγ produced by primary mouse CD8^+^ T cells was measured as described above using 1 µg/mL capture antibody (clone XMG1.2) and 0.4 µg/mL biotinylated antibody (clone R4-6A2). Sample IFNγ concentration was determined using recombinant IFNγ (eBioscience) as a standard.

IL-2 produced by NOD-derived T cell hybridomas was measured using mouse Cytokine Panel 6 plates from Meso Scale Discovery (Rockville, MD) as per manufacturer instructions.

### Immunoblotting

Cell lysates for analysis of neddylated proteins were obtained by resuspending cells in lysis buffer containing 15 mM Tris base, 0.5 M NaCl, 0.35% Nonidet P-40 substitute (NP-40) (Sigma-Aldrich), plus inhibitors as previously published [24]. OPT (2 mM) was also added to the lysis buffer as a non-specific inhibitor of CSN proteases [25]. The resulting lysates were denatured with an equal volume of 2 X Laemmli sample buffer and incubated at 95°C for 10 minutes. Immunoblots were performed as per our previously published methods [26]. Chemiluminescent signal was detected using Pierce ECL Western Blotting Substrate (Thermo Fisher) and GeneMate Blue Basic Autorad film (BioExpress). Immunoblot band relative density was quantified and normalized to the density of the loading control using ImageJ software (Research Services Branch, National Institute of Mental Health).

### Cell Death Analysis

The MA5.8ζ T cell hybridoma line was cultured with varying concentrations of MLN4924 for 48 hours. Cells were stained with annexin V (eBioscience) and propidium iodide (eBioscience) according to manufacturer instructions. Data were collected on a CyAn flow cytometer (Dako) within 1 hour of staining and analyzed using FlowJo v.8.7.1 software (Tree Star).

### Statistical Analysis

Unpaired two-tailed Student t tests were performed using Prism 5.0a (GraphPad Software). Differences were considered statistically significant for p values <0.05.

## Results and Discussion

### Neddylation Sets the Threshold for TCR Complex-mediated Cytokine Production, Proliferation, and Effector Differentiation

**Figure 1:**
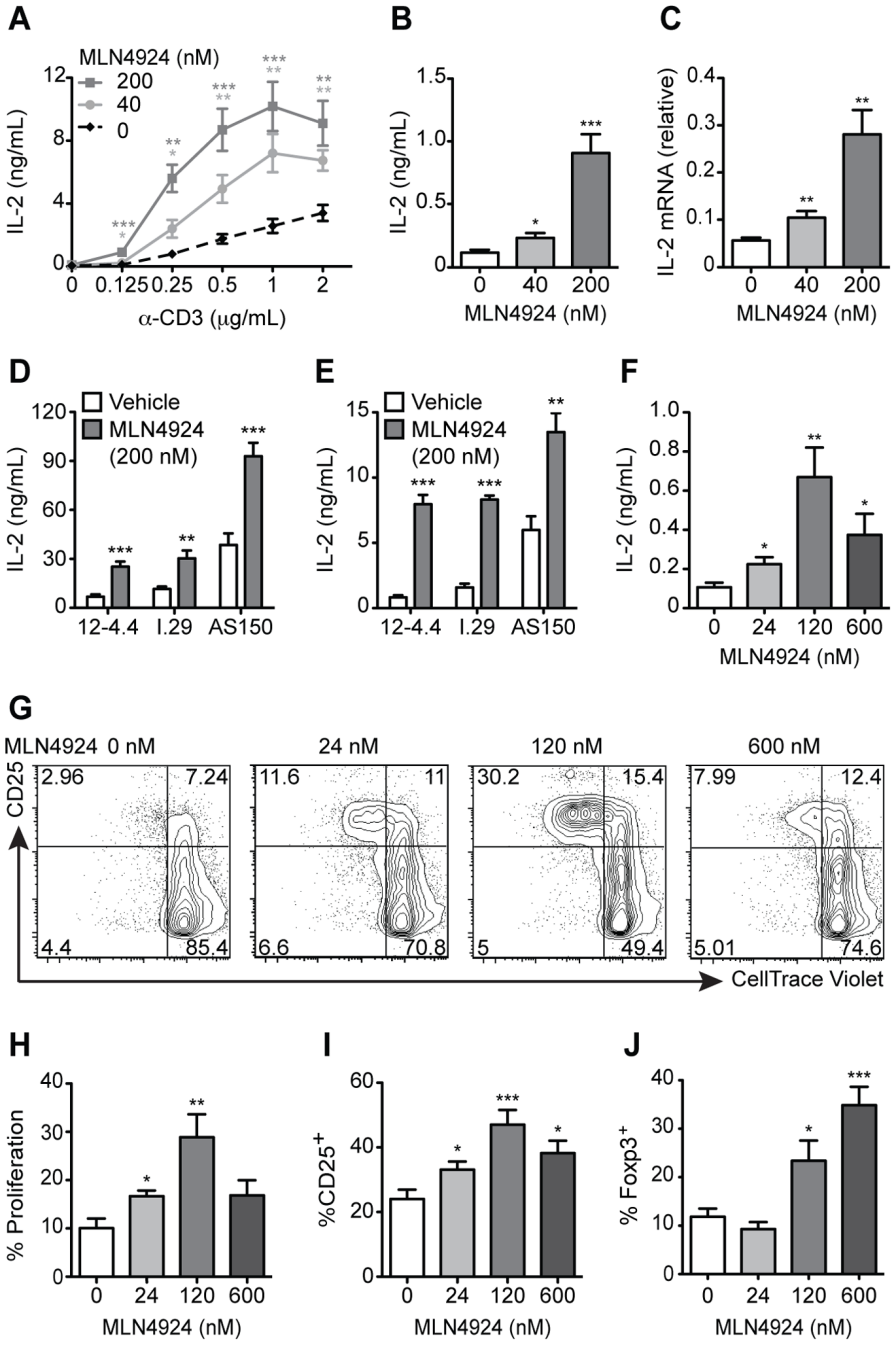
MLN4924 lowers the threshold for T cell cytokine production, proliferation and effector differentiation. (A) IL-2 secretion by MA5.8ζ T cells after 48 h of α-CD3 and MLN4924 at the indicated concentrations. Data averaged from three to four independent experiments. (B) MA5.8ζ IL-2 secretion at 48 h (n = 3) and (C) transcription at 24 h with 0.125 µg/mL α-CD3 and MLN4924 (n = 3). (D) IL-2 secreted by the NOD-derived T cell hybridomas 12-4.4, I.29 and AS150 cultured with 1 µg/mL α-CD3 (n = 3) or (E) with live M12C3G7 APCs presenting the insulin peptide B:9–23 and MLN4924 after 24 h (n = 3). (F) IL-2 secreted by primary CD4^+^ T cells after stimulation with 1 µg/mL α-CD3 and MLN4924 at 72 h (n = 4). (G) Representative flow cytometry plots of CD25 expression and CellTrace Violet dilution in primary CD4^+^ T cells cultured as described in (F). (H) Percent proliferation of CD4^+^ T cells cultured as in (F) (n = 3). (I) Percent CD25^+^ of CD4^+^ T cells cultured as in (F) (n = 4). (J) Percent SKG Foxp3^+^ iTregs after culturing CD4^+^CD25^−^ T cells for 96 h in polarizing conditions with 2.5 µg/mL α-CD3 and MLN4924 (n = 3). Values represent mean ± s.e.m.; *P<0.05, **P<0.01, ***P<0.001.

To investigate the contribution of neddylation in regulating TCR complex-mediated signaling, T cells were treated with MLN4924, using doses known to inhibit neddylation [27]. The MA5.8 T cell hybridoma, which expresses TCRζ from an exogenous promoter, was used because it produces higher levels of TCR leading to a greater dynamic range of IL-2 production upon TCR stimulation [15], [16]. These cells were treated with increasing concentrations of both α-CD3 and MLN4924. Up to 200 nM MLN4924 increased MA5.8ζ interleukin 2 (IL-2) production. At the 200 nM MLN4924 dose, enhancement of IL-2 ranged from 8-fold using low concentrations of α-CD3 (0.125 µg/ml; Fig. 1A, 1B) to 3-fold with higher concentrations of α-CD3 (2 µg/ml; Fig. 1A). This indicates that inhibiting neddylation lowers the threshold of TCR stimulation for IL-2 production. Using higher concentrations of MLN4924 (1 µM) increased toxicity, which was demonstrated by a reduction in cell survival based on annexin V and propidium iodide staining (Figure S1A). The observed toxicity is consistent with known effects of MLN4924 on arresting the cell cycle [14]. Furthermore, the increase in IL-2 secretion seen with non-toxic MLN4924 levels was due, at least in part, to elevated levels of IL-2 transcript (Fig. 1C).

To determine whether the increased IL-2 secretion seen with neddylation inhibition occurred upon antigen-specific TCR stimulation, we used a panel of insulin specific T cell hybridomas derived from NOD mice. The hybridomas have established differences in IL-2 responses to the register-trapped peptide B:9–22(RE) presented in the context of murine MHC class II molecule I-A^g7^ on antigen presenting cells (APCs) [17]. In concordance with previously published results, the 12-4.4 hybridoma produced the least amount of IL-2 without MLN4924 treatment, while the AS150 hybridoma produced the most (Fig. 1D, 1E) [17]. Addition of 200 nM MLN4924 increased IL-2 production in all three hybridomas regardless of whether the T cells were stimulated with α-CD3, live or fixed B:9–22(RE) APCs expressing B:9–22(RE) covalently linked to I-A^g7^ (Fig. 1D, 1E; and Figure S1B). These studies demonstrate, in four different T cell hybridomas, that MLN4924 enhances TCR complex-mediated IL-2 production and decreases the amount of stimulatory input required for robust signaling.

To determine whether inhibition of neddylation would increase IL-2 secretion in primary T cells, CD4^+^ T cells were isolated from BALB/c mice and stimulated with either low (1 µg/mL) or high (4 µg/mL) doses of α-CD3, in the presence or absence of MLN4924 (Fig. 1F; and Figure S1C). To generate detectable amounts of IL-2 in primary CD4^+^ T cells, higher concentrations of α-CD3 were required. CD4^+^ T cells stimulated with low dose α-CD3 in the presence of 24, 120, and 600 nM MLN4924 had IL-2 production increased by 2-, 6-, and 3-fold respectively (Fig. 1F). Moreover, the treated primary CD4^+^ T cells treated with 120 nM MLN4924 demonstrated increased T cell activation, as evidenced by a 2–3-fold increase in both proliferation and CD25 expression (Fig. 1G–1I). However, the increase was blunted with 600 nM MLN4924 (Fig. 1G–1I), correlating with diminished IL-2 production using the same conditions (Fig. 1F). In contrast, stimulation of primary CD4^+^ T cells with 4 µg/mL of α-CD3 and increasing amounts of MLN4924 reduced IL-2 production in a dose-dependent manner (Figure S1C). Thus, while MLN4924 decreased the required amount of TCR signal required for IL-2 production in primary cells, the toxic effects of MLN4924 became more pronounced with higher amounts of TCR stimulation.

Similar to primary CD4^+^ T cells, 120 nM MLN4924 treatment of primary CD8^+^ T cells from C57BL/6 (B6) mice also showed increased production of the cytokine interferon gamma (IFNγ at low dose α-CD3 stimulation (0.5 µg/mL), which was abrogated with high dose α-CD3 stimulation (2 µg/mL) (Figure S1D, S1E). These results are consistent with a recently published study demonstrating that treatment of primary CD4^+^ T cells with high dose α-CD3 and α-CD28 stimulation, in conjunction with MLN4924, decreased T cell proliferation, activation marker induction, and IL-2 production [28]. As this study only used high dose stimulation, the ability of MLN4924 to enhance TCR complex-mediated IL-2 production was not observed. Further, our results are consistent with the observation that primary CD4^+^ T cells with increased neddylation, due to deficiency in the COP9 signalosome subunit 8 involved in deneddylation, had reduced IL-2 production [29].

To test if MLN4924 could modulate T cell effector differentiation, purified resting CD4^+^CD25^−^ T cells from BALB/c and SKG mice, which have a defect in TCR signaling through ZAP70 [30], were stimulated with low dose α-CD3 (0.5 µg/mL for BALB/c and 2.5 µg/mL for SKG) and increasing doses of MLN4924 in conditions that favor the development of inducible regulatory T cells (iTregs) [23]. MLN4924 treatment enhanced the production of iTregs in a dose-dependent manner, supporting the notion that neddylation status modulates T cell effector differentiation (Figure S1F; Fig. 1J). In summary, inhibition of neddylation by MLN4924 increased TCR-stimulated cell proliferation, IL-2 production, and iTreg development.

### Loss of CUL Neddylation Upon TCR Stimulation

**Figure 2:**
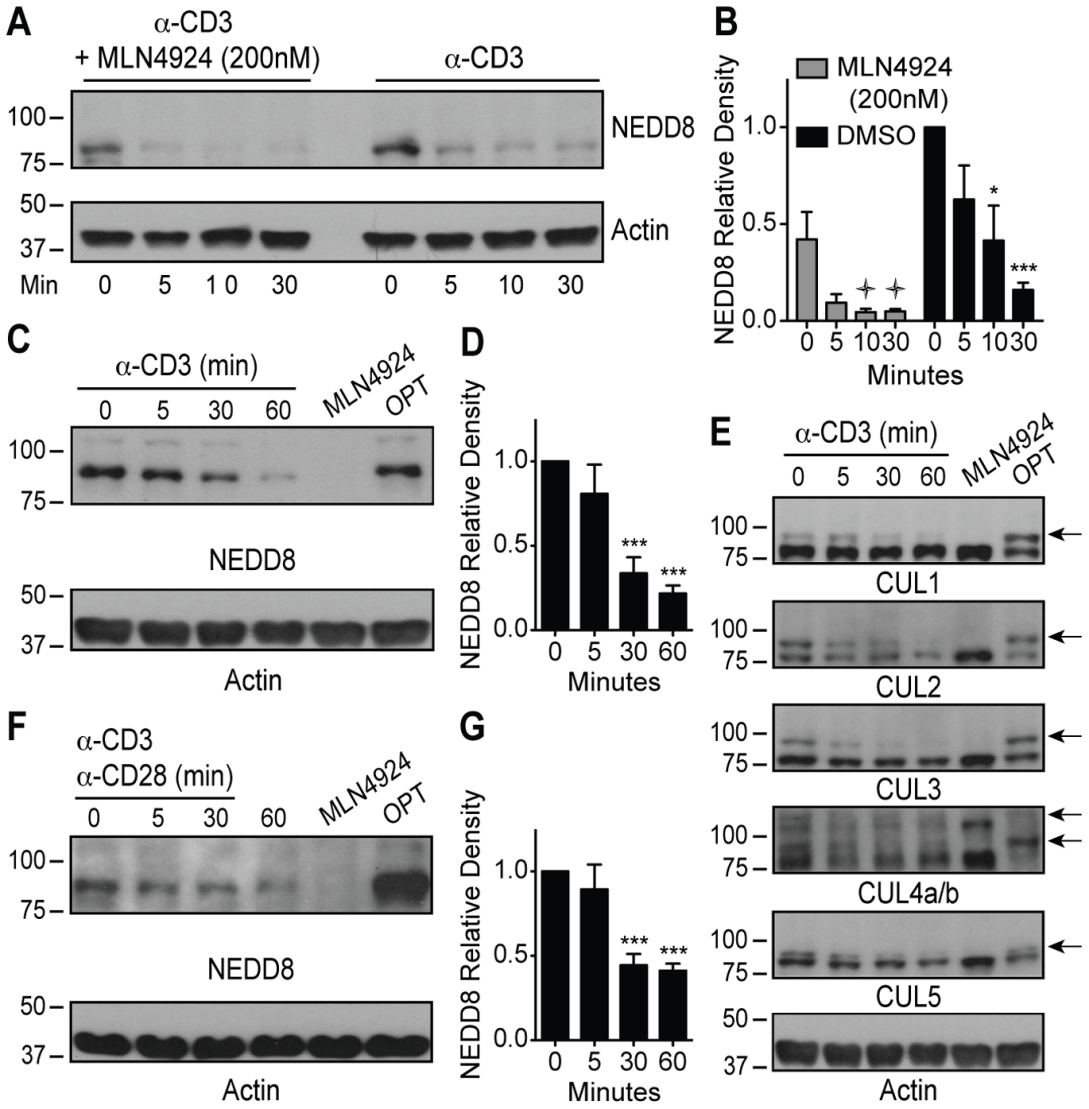
TCR complex-mediated signaling reduces CRL neddylation. (A) Representative immunoblots and (B) quantification of NEDD8 relative density from MA5.8ζ cells stimulated with 5 µg/mL α-CD3 with or without 200 nM MLN4924 (n = 4); 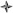
P<0.05 compared to 0 min with MLN4924. (C) Representative immunoblots and (D) quantification of NEDD8 relative density from primary CD4^+^ T cells stimulated with 5 µg/mL α-CD3 or incubated with either 3 µM MLN4924 for 2 h as a deneddylated control or 2 mM OPT for 30 min as a neddylated control (n = 4). (E) Representative cullin immunoblots from primary CD4^+^ T cells stimulated as in (C) (arrows indicate neddylated CULs) (n = 3). (F) Representative immunoblots and (G) quantification of NEDD8 relative density from human cord blood CD4^+^ T cells stimulated with 10 µg/mL α-CD3 and 2 µg/mL α-CD28 or incubated either with 3 µM MLN4924 for 2 h or 2 mM OPT for 30 min (n = 4). Values represent mean ± s.e.m.; *P<0.05, **P<0.01, ***P<0.001 compared to 0 min.

To determine if TCR stimulation alters protein neddylation and identify the neddylated proteins responsible for regulating TCR complex-mediated signaling, we pretreated MA5.8ζ T cells with either 200 nM MLN4924 or vehicle control, and stimulated the cells with 5 µg/mlα-CD3 stimulation for 0, 5, 10, and 30 minutes. Cell lysates were immunoblotted with a NEDD8-specific antibody (Fig. 2A, 2B) to identify neddylated proteins. MLN4924 treatment alone (time 0) decreased neddylation of proteins in the 80–95 kDa range, and neddylation of these proteins decreased further upon TCR complex-mediated stimulation. Surprisingly, the same phenomenon was observed in the vehicle only-treated cells, revealing that TCR stimulation induces loss of neddylation of these proteins. To demonstrate that loss of neddylation of the 80–95 kDa proteins was not limited to a T cell hybridoma (MA5.8ζ), primary CD4^+^ T cells were stimulated with α-CD3 for 5, 30, and 60 minutes to generate cell lysates for immunoblot with an NEDD8-specific antibody (Fig. 2C, 2D). Treatment with high dose (3 µM) MLN4924 served as a negative neddylation control, and treatment with 2 mM o-phenanthroline (OPT), an inhibitor of the COP9 signalosome, served as a positive control for neddylation [31]. Similar to the MA5.8ζ T cells, primary mouse CD4^+^ T cells also had a loss of neddylation of 80–95 kDa proteins upon TCR stimulation. Previous work has shown that six CULs co-migrate between 85–110 kDa: CUL1, CUL2, CUL3, CUL4a, CUL4b, and CUL5, and that CUL neddylation is inhibited by MLN4924 [32]. Thus, specific immunoblots for these CULs were performed (Fig. 2E). Loss of CUL neddylation can be detected through a mass shift [31]. CUL specific immunoblots demonstrated that upon TCR stimulation, all CULs, which are neddylated at baseline, lose at least some neddylation (Fig. 2E).

To establish if human CD4^+^ T cells also have coordinated loss of CUL neddylation upon TCR stimulation, purified human cord blood CD4^+^ T cells were stimulated with α-CD3 and α-CD28. Again, loss of CUL neddylation, as evident by total NEDD8 immunoblot, was noted upon TCR stimulation (Fig. 2F, 2G). These studies demonstrate that, in both murine and human CD4^+^ T cells, TCR stimulation results in loss of CUL neddylation.

To determine if the loss of CUL neddylation was restricted to CD4^+^ T cells, similar studies were performed using primary B6 mouse CD8^+^ T cells. Indeed, α-CD3 stimulation triggered loss of CUL neddylation, albeit with slightly different kinetics and magnitude of response (Figure S2A, S2B).

### Loss of CUL1, CUL2 and CUL3 Lead to Increased IL-2 Production Upon TCR Stimulation

**Figure 3:**
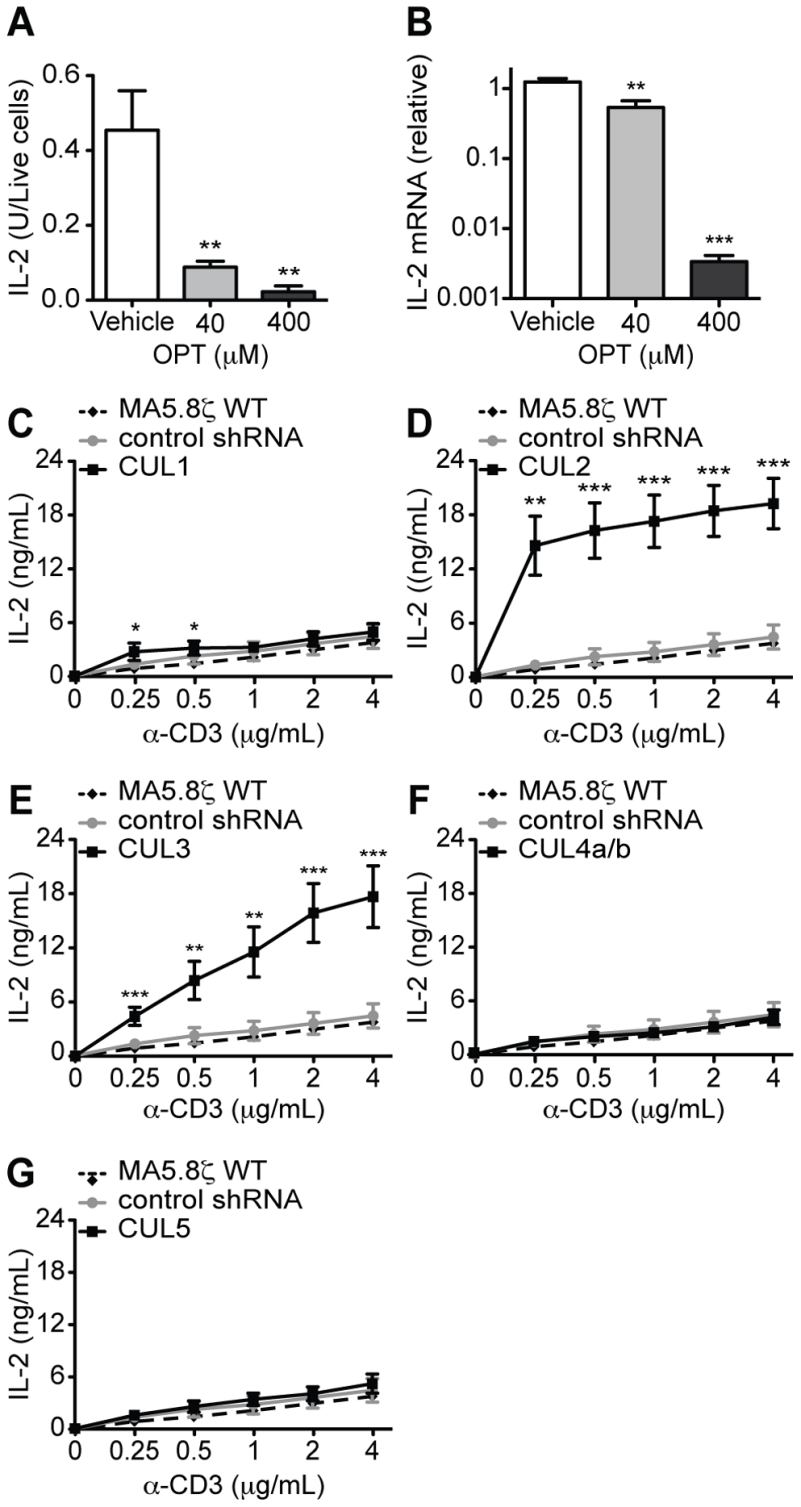
Activated CRLs containing CUL1 and especially CUL2 and CUL3 regulate IL-2 production. (A) MA5.8ζ IL-2 secretion at 12 h normalized to the number of live cells measured by annexin V and propidium iodide staining and (B) transcription at 12 h with 4 µg/mL α-CD3 and the indicated concentrations of OPT (n = 3). (C–G) IL-2 secretion by MA5.8ζ cells, cells expressing control shRNA, and cells expressing an shRNA for (C) CUL1, (D) CUL2, (E) CUL3, (F) CUL4a/b and (G) CUL5 after 48 h of stimulation with indicated concentrations of α-CD3. Data are averaged from two independent experiments, where each experiment examined three independently created lines for each shRNA. (H) IL-2 secretion at 48 h by MA5.8ζ cells and cells expressing either control shRNA, CUL2 shRNA or CUL3 shRNA with 1 µg/mL α-CD3 and 200 nM MLN4924. Data are averaged as in (C–G). Values represent mean ± s.e.m.; *P<0.05, **P<0.01, ***P<0.001.

To determine if loss of CUL neddylation and CRL activity is responsible for alterations in IL-2 production seen in MLN4924-treated T cells, the deneddylase inhibitor OPT was used to determine if maintenance of protein neddylation inhibits IL-2 production upon TCR stimulation (Fig. 3A). While OPT reduced the number of live cells in culture, possibly due to off target effects as a zinc chelator and inhibitor of various metalloproteases, treatment of MA5.8ζ T cells with increasing doses of OPT in the presence of optimal TCR stimulation led to a significant decrease in both IL-2 protein and transcript levels relative to the number of live cells, and IL-2 transcript (Fig. 3A, 3B). These studies show that forced maintenance of protein neddylation inhibits TCR complex-mediated IL-2 production.

To determine if loss of specific CUL neddylation and CRL activity is responsible for the alterations in IL-2 production, each CUL gene was knocked down in MA5.8ζ T cells, and IL-2 production was measured. Knockdown cell lines were generated using lentiviral vectors containing either a control shRNA for green fluorescent protein (GFP) or shRNA for each CUL, achieving between 71–96% protein knockdown (Figure S3A–S3E). The shRNA for CUL4a also knocked down the CUL4b isoform. Complete elimination of CUL expression and CRL activity was likely not feasible, as CRLs are required to regulate many cellular functions, and gene knockouts of CULs are lethal or lead to cell cycle arrest in multiple models [9].

CUL knockdown lines were stimulated with increasing amounts of α-CD3 to reveal any changes in signaling thresholds. Expression of a control shRNA specific for GFP did not alter the responsiveness of MA5.8ζ T cells to α-CD3 stimulation when compared to the non-transfected parental control (Fig. 3C–3G). In contrast, despite incomplete knockdown, decreased CUL1, CUL2, and CUL3 expression led to increased IL-2 production (Fig. 3C–3F). CUL1 knockdown had a modest increase in IL-2 production seen only at low levels of α-CD3 stimulation. However, CUL2 knockdown led to a robust increase in IL-2 production (∼15 fold) at all doses of TCR stimulation. CUL3 knockdown also led to an almost 15-fold increase in IL-2 production, but only at higher TCR stimulation. The lack of an effect in the CUL4a/b and CUL5 knockdowns demonstrates specificity in the system. This suggests that proteins ubiquitinated by CUL2 and CUL3 derived CRLs have the most involvement in regulating TCR complex-mediated IL-2 production.

Recently, CUL3 was shown to associate with promyelocytic leukaemia zinc finger (PLZF), a transcription factor required for the development of invariant natural killer T cells (iNKT) [33]. T cell-specific deletion of CUL3 led to a defect in iNKT cell development [33]. However, the contribution of CUL3 to the regulation of conventional CD4 and CD8 T cell activation as well as IL-2 production upon TCR stimulation was not reported. Our studies provide new and compelling evidence that CRLs derived from predominately CUL2, CUL3, and potentially CUL1 are important negative regulators of specific downstream actions of TCR complex signaling.

### Global Mechanism of CUL Deneddylation Upon Tyrosine Kinase-based Receptor Signaling

To determine if CUL deneddylation serves as a more universal mechanism to regulate tyrosine kinase-based receptor signaling, we investigated a number of different cell types. As the B cell receptor (BCR) initiates a similar tyrosine kinase-based signaling cascade [6], purified splenic B cells from BALB/c mice were stimulated through the BCR signaling complex using an α-IgM F(ab’)_2_ (Figure S4A, S4B). Consistent with the T cell data, B cells had a loss of CUL neddylation upon BCR complex-mediated signaling. However, in contrast to T cells, B cells had a transient increase in CUL neddylation that is gone by 30 minutes of stimulation. The importance of this initial increase in CUL neddylation in B cells is unknown but reinforces that despite sharing many features, the BCR and TCR each activate specific signaling networks.

**Figure 4:**
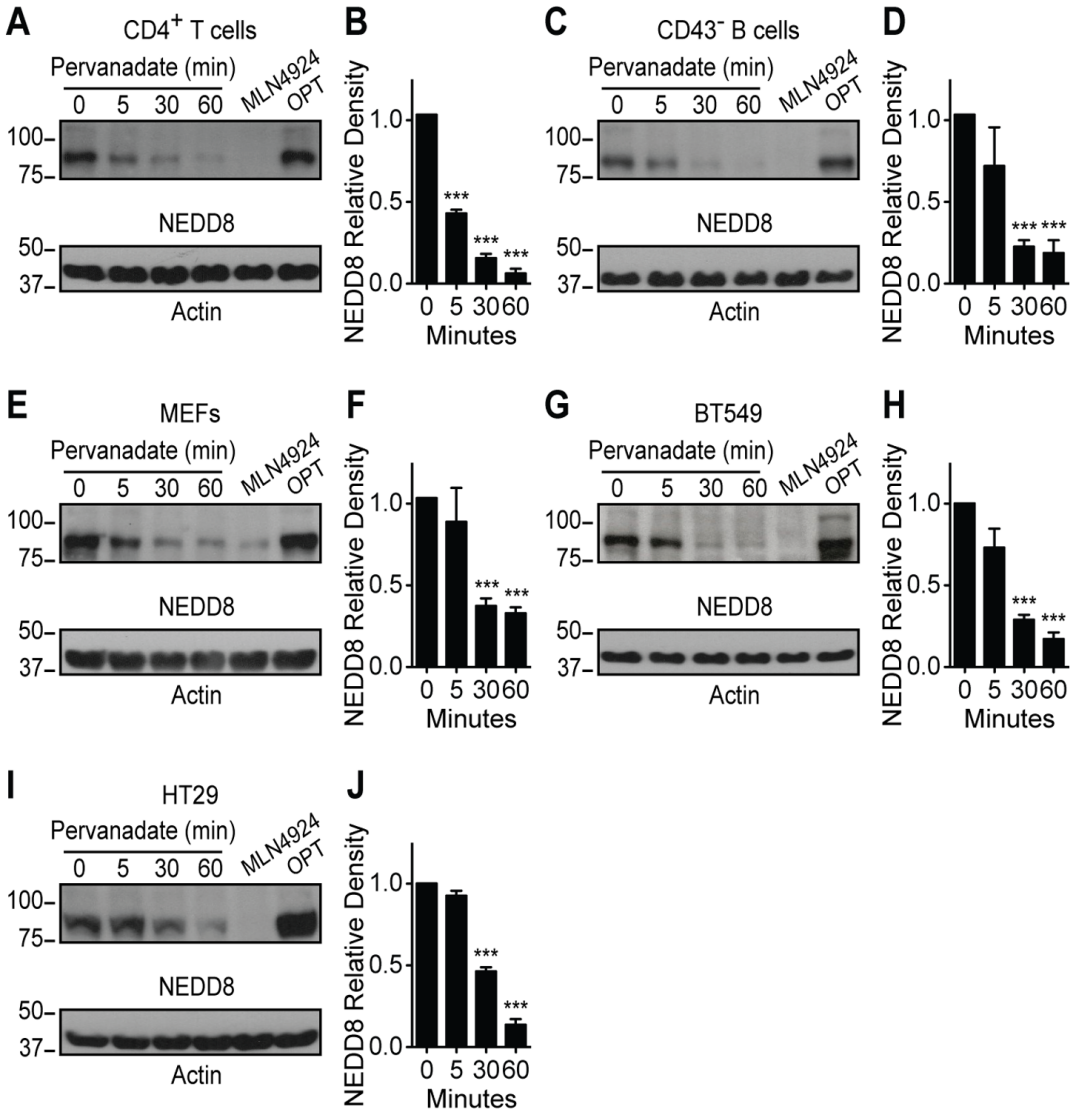
Pervanadate stimulation reduces CRL neddylation. (A) Representative immunoblot and (B) quantification of NEDD8 relative density from primary mouse CD4^+^ T cells stimulated with 100 µM pervanadate or incubated either with 3 µM MLN4924 for 2 h as a dennedylated control or 2 mM OPT for 30 min as a neddylated control (n = 3). (C) Representative immunoblot and (D) quantification of NEDD8 relative density from primary mouse CD43^−^ B cells stimulated as in (B) (n = 3). (E) Representative immunoblot and quantification (F) of NEDD8 relative density from MEFs stimulated as in (B) (n = 3). (G) Representative immunoblot and (H) quantification of NEDD8 relative density from BT549 cells stimulated as in (B) (n = 3). (I) Representative immunoblot and (J) quantification of NEDD8 relative density from HT29 colon cancer cells stimulated as in (B) (n = 3). Values represent mean ± s.e.m.; *P<0.05, **P<0.01, ***P<0.001.

To establish if strong tyrosine kinase-based signals can lead to loss of CUL neddylation in other cells types, an inhibitor of tyrosine phosphatases, pervanadate, was employed to determine if other tyrosine kinase-based signals lead to CUL deneddylation [34]. Initially mouse CD4^+^ T cells and splenic B cells were treated with pervanadate as controls. Loss of CUL neddylation was seen in both cell types upon pervanadate treatment, but the kinetics and magnitude of response was greater with pervanadate than antigen receptor-specific stimulation (Fig. 4A–4D). In addition, the transient increase in CUL neddylation seen in α-IgM treated B cells was lost upon pervanadate treatment (Fig. 4C, 4D). Thus, a robust unopposed tyrosine kinase-based signal, achieved through the inhibition of tyrosine phosphatases, can lead to loss of CUL neddylation in T and B cells. Next, primary mouse embryonic fibroblasts (MEFs), a breast cancer cell line (BT549), and a colon cancer cell line (HT29) were treated with pervanadate, and CUL neddylation status was determined. Pervanadate reduced CUL neddylation by at least 50% within 30 minutes of treatment in all three cell lines (Fig. 4E–4J). While other studies have shown that signaling through the G-protein coupled receptors, adenosine A2B receptor and adrenomedullin receptor, led to loss of CUL1 and CUL2 neddylation [35], [36], our studies are the first to show that tyrosine kinase-based signaling can globally regulate the level of CUL neddylation.

Based on our results, we propose that a reciprocal relationship exists between tyrosine kinase-based signaling and CUL neddylation. Neddylated CRLs prevent low-level stimulation from initiating a proliferative response, while strong stimulation abrogates CRL activity to prevent degradation of proteins essential for signaling. In demonstrating this relationship between TCR complex signaling and neddylation of CRLs, our findings present a new paradigm in the regulation of T cell signaling thresholds.

More than 16 drugs targeting kinases or phosphatases are approved for patients, with another 150 in early trials. Although the number of ubiquitin ligases and deubiquitinases expressed in T cells is similar to the number of kinases and phosphatases, only 2 drugs that target the ubiquitin pathway are approved, bortezomib (VELCADE®) and carfilzomib (Kyrprolis®), while 16 are in early trials [37], [38]. As our studies provide insight regarding the ubiquitin-dependent mechanisms that globally regulate tyrosine kinase-based signaling, further investigation will be required to understand how to specifically modulate this pathway. Further understanding of which adaptors are utilized by CRLs and which proteins are targeted for ubiquitination in TCR and BCR signaling will enable development of more targeted strategies to treat immune-mediated disease.

## Supporting Information

Figure S1 (A) Gating strategy, percent live cells and absolute number of live MA5.8ζ cells after incubating for 48 h with MLN4924 at the indicated concentrations. Doublets were first excluded before gating on intact cells and then cells live cells that did not stain with annexin V or propidium iodide. The absolute number of live cells was determined by multiplying cell count with the percent of live cells (n = 3± s.e.m.). (B) IL-2 secreted by the NOD-derived T cell hybridomas, I.29 and AS150, cultured with fixed M12C3-B:9–22(RE) APCs and MLN4924 after 24 h (n = 2± s.e.m.). (C) IL-2 secreted by primary BALB/c mouse CD4^+^ T cells after stimulation with 4 µg/mL α-CD3 and MLN4924 at 72 h (n = 4± s.e.m.). (D) IFNγ secreted by primary B6 mouse CD8^+^ T cells after stimulation with 0.5 µg/mL α-CD3 and MLN4924 at 72 h (n = 3± s.e.m.). (E) IFNγ secreted by primary B6 mouse CD8^+^ T cells after stimulation with 2 µg/mL α-CD3 and MLN4924 at 72 h (n = 3± s.e.m.). (F) Percent BALB/c Foxp3^+^ iTregs after culturing mouse CD4^+^CD25^−^ T cells for 96 h in polarizing conditions with 0.5 µg/mL α-CD3 and MLN4924 (n = 3±s.e.m.).
(TIF)

Figure S2 (A) Representative immunoblot and (B) quantification of NEDD8 relative density from primary B6 mouse CD8^+^ T cells stimulated with 5 µg/mL α-CD3 or incubated with either 3 µM MLN4924 for 2 h as a dennedylated control or 2 mM OPT for 30 min as a neddylated control (n = 2± s.e.m.).
(TIF)

Figure S3 (A–E) Representative immunoblot of cullins from MA5.8ζ cells, cells expressing control shRNA and the three bulk cell lines expressing an shRNA for (A) CUL1, (B) CUL2, (C) CUL3, D (CUL4a/b) and (E) CUL5.
(TIF)

Figure S4 (A) Representative immunoblot and (B) quantification of NEDD8 relative density from primary BALB/c mouse CD43^−^ B cells stimulated with 20 µg/mL α-IgM or incubated with either 3 µM MLN4924 for 2 h as a dennedylated control or 2 mM OPT for 30 min as a neddylated control (n = 2± s.e.m.).
(TIF)
