# Supplementary figures and images for: The Discovery of a Reciprocal Relationship between Tyrosine-Kinase Signaling and Cullin Neddylation
Source: PLoS One. 2013 Oct 4;8(10):e75200. doi: 10.1371/journal.pone.0075200 (PMC3790728; doi:10.1371/journal.pone.0075200)

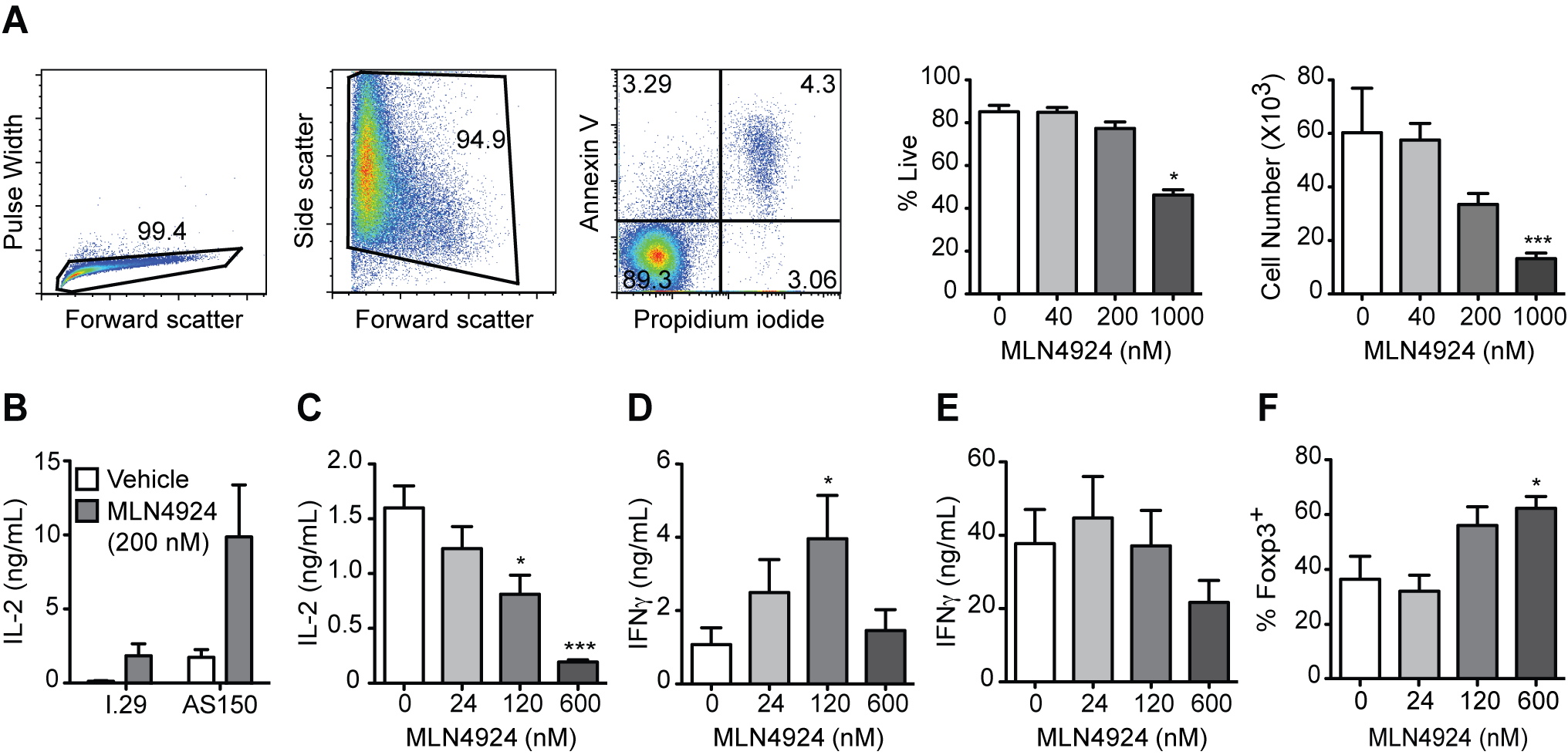

Supplement: Figure S1 — (A) Gating strategy, percent live cells and absolute number of live MA5.8ζ cells after incubating for 48 h with MLN4924 at the indicated concentrations. Doublets were first excluded before gating on intact cells and then cells live cells that did not stain with annexin V or propidium iodide. The absolute number of live cells was determined by multiplying cell count with the percent of live cells (n = 3± s.e.m.). (B) IL-2 secreted by the NOD-derived T cell hybridomas, I.29 and AS150, cultured with fixed M12C3-B:9–22(RE) APCs and MLN4924 after 24 h (n = 2± s.e.m.). (C) IL-2 secreted by primary BALB/c mouse CD4+ T cells after stimulation with 4 µg/mL α-CD3 and MLN4924 at 72 h (n = 4± s.e.m.). (D) IFNγ secreted by primary B6 mouse CD8+ T cells after stimulation with 0.5 µg/mL α-CD3 and MLN4924 at 72 h (n = 3± s.e.m.). (E) IFNγ secreted by primary B6 mouse CD8+ T cells after stimulation with 2 µg/mL α-CD3 and MLN4924 at 72 h (n = 3± s.e.m.). (F) Percent BALB/c Foxp3+ iTregs after culturing mouse CD4+CD25− T cells for 96 h in polarizing conditions with 0.5 µg/mL α-CD3 and MLN4924 (n = 3±s.e.m.). (TIF) [file pone.0075200.s001.tif]

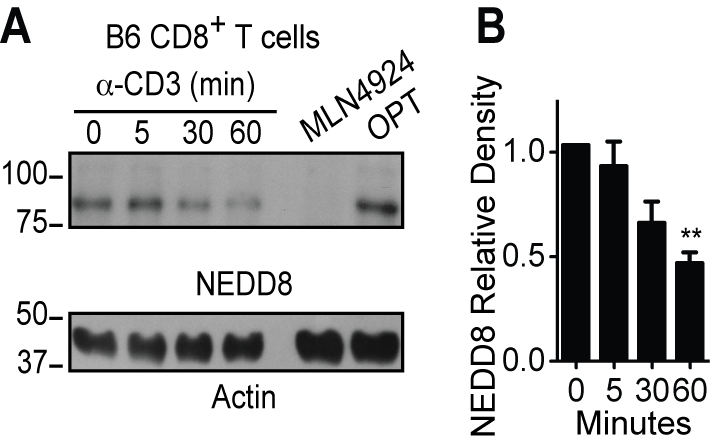

Supplement: Figure S2 — (A) Representative immunoblot and (B) quantification of NEDD8 relative density from primary B6 mouse CD8+ T cells stimulated with 5 µg/mL α-CD3 or incubated with either 3 µM MLN4924 for 2 h as a dennedylated control or 2 mM OPT for 30 min as a neddylated control (n = 2± s.e.m.). (TIF) [file pone.0075200.s002.tif]

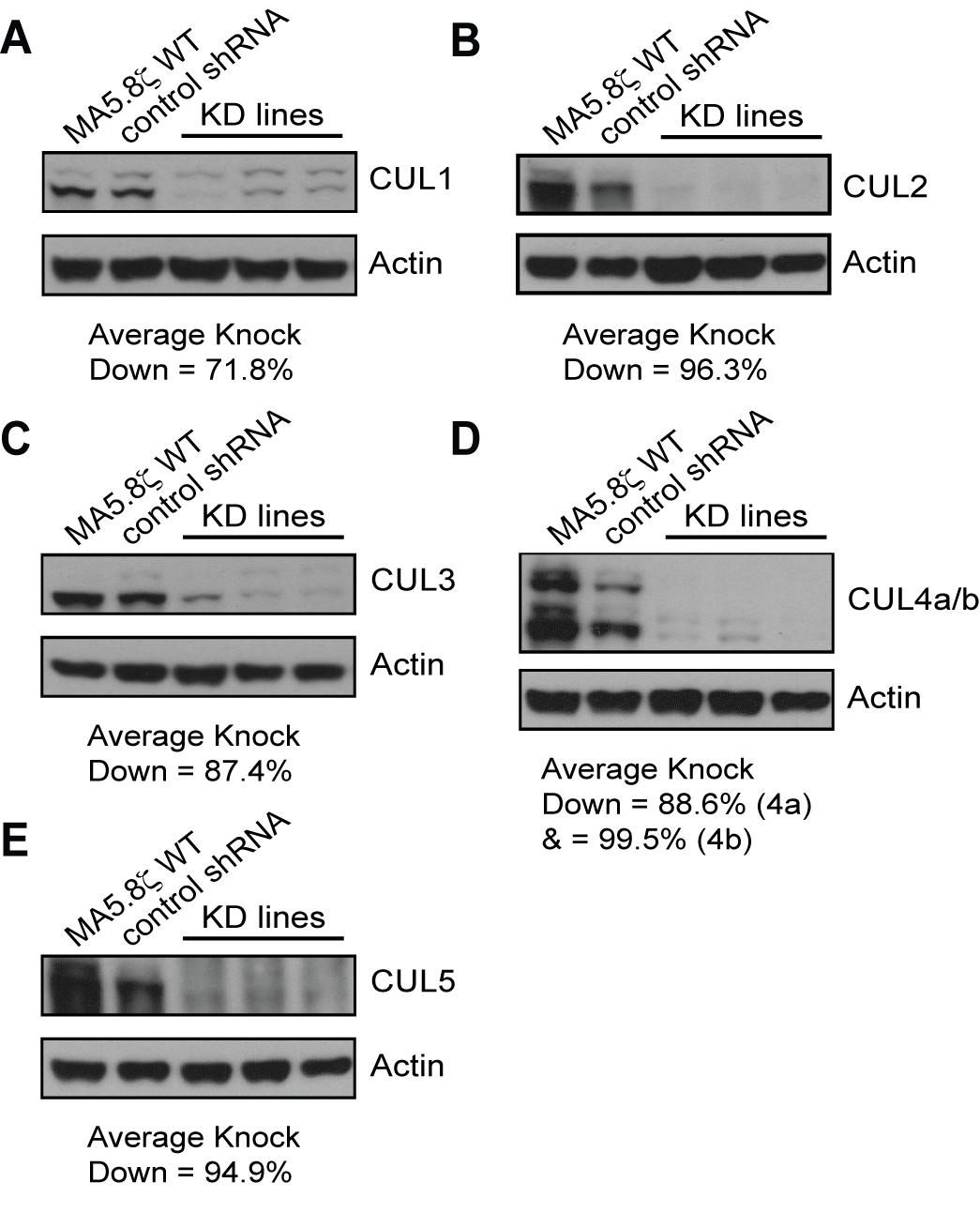

Supplement: Figure S3 — (A–E) Representative immunoblot of cullins from MA5.8ζ cells, cells expressing control shRNA and the three bulk cell lines expressing an shRNA for (A) CUL1, (B) CUL2, (C) CUL3, D (CUL4a/b) and (E) CUL5. (TIF) [file pone.0075200.s003.tif]

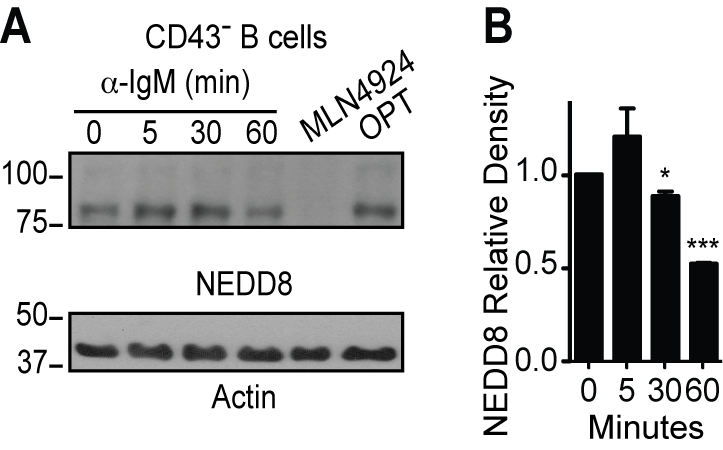

Supplement: Figure S4 — (A) Representative immunoblot and (B) quantification of NEDD8 relative density from primary BALB/c mouse CD43− B cells stimulated with 20 µg/mL α-IgM or incubated with either 3 µM MLN4924 for 2 h as a dennedylated control or 2 mM OPT for 30 min as a neddylated control (n = 2± s.e.m.). (TIF) [file pone.0075200.s004.tif]
